# Nicotinamide N-Methyltransferase in Head and Neck Tumors: A Comprehensive Review

**DOI:** 10.3390/biom11111594

**Published:** 2021-10-28

**Authors:** Lucrezia Togni, Marco Mascitti, Davide Sartini, Roberto Campagna, Valentina Pozzi, Eleonora Salvolini, Annamaria Offidani, Andrea Santarelli, Monica Emanuelli

**Affiliations:** 1 Department of Clinical, Specialistic and Dental Sciences, Marche Polytechnic University, 60126 Ancona, Italy; togni.lucrezia@gmail.com (L.T.); m.mascitti@staff.univpm.it (M.M.); d.sartini@staff.univpm.it (D.S.); r.campagna@univpm.it (R.C.); v.pozzi@staff.univpm.it (V.P.); e.salvolini@univpm.it (E.S.); m.emanuelli@staff.univpm.it (M.E.); 2 Department of Clinical and Molecular Sciences, Marche Polytechnic University, 60126 Ancona, Italy; a.m.offidani@univpm.it; 3 Dentistry Clinic, National Institute of Health and Science of Aging, IRCCS INRCA, 60126 Ancona, Italy

**Keywords:** nicotinamide N-methyltransferase, nicotinamide, head and neck tumor, oral squamous cell carcinoma, esophageal squamous cell carcinoma, thyroid cancer, diagnostic marker, prognosis

## Abstract

The head and neck tumors (HNT) are a heterogeneous group of diseases ranging from benign to malignant lesions, with distinctive molecular and clinical behaviors. Several studies have highlighted the presence of an altered metabolic phenotype in HNT, such as the upregulation of nicotinamide N-methyltransferase (NNMT). However, its biological effects have not been completely disclosed and the role of NNMT in cancer cell metabolism remains unclear. Therefore, this comprehensive review aims to evaluate the available literature regarding the biological, diagnostic, and prognostic role of NNMT in HNT. NNMT was shown to be significantly overexpressed in all of the evaluated HNT types. Moreover, its upregulation has been correlated with cancer cell migration and adverse clinical outcomes, such as high-pathological stage, lymph node metastasis, and locoregional recurrences. However, in oral squamous cell carcinoma (OSCC) these associations are still debated, and several studies have failed to demonstrate the prognostic significance of NNMT. The shRNA-mediated gene silencing efficiently suppressed the NNMT gene expression and exhibited a clear inhibitory effect on cell proliferation, promoting the expression of apoptosis-related proteins and modulating the cell cycle. NNMT could represent a new molecular biomarker and a new target of molecular-based therapy, although further studies on larger patient cohorts are needed to explore its biological role in HNT.

## 1. Introduction

The head and neck tumors (HNT) are a heterogeneous group of lesions with different embryological and histological origins that range from benign to malignant entities, arising from tissues of the head and neck region [1]. The predominant type of HNT consists of squamous cell carcinomas arising from oral, oropharyngeal, nasal cavities, paranasal sinuses, pharynx, and larynx, collectively defined as head and neck squamous cell carcinomas (HNSCC) [2]. Within the HNT group, the salivary gland tumors, a group of lesions that are characterized by heterogeneous histopathological and clinical presentations, and odontogenic tumors, a group of hard tissue neoplasms derived from the tooth-forming apparatus are also included [3]. Other tumor types, such as mucosal melanomas, esophageal, and thyroid cancers are not universally accepted as part of HNT. The main reason is the lack of a strict definition of HNT that is universally accepted. Indeed, HNT are highly heterogeneous in terms of epidemiology, risk factors, clinical manifestations, and prognosis. Therefore, the range of tumors that are included within this group may vary depending on which source is considered [4]. Several studies have highlighted the presence of an altered metabolic phenotype in HNT cells that is a consequence of oncogenic processes. Since an altered metabolism is an important component of tumorigenesis and disease progression, it could be a source of diagnostic and prognostic markers, as well as a promising opportunity for drug therapy [5]. Among the studies that focused on specific metabolic pathway alterations, attention has been focused on the enzymes that are involved in the nicotinamide metabolism, including the nicotinamide N-methyltransferase (NNMT).

NNMT (EC 2.1.1.1.) is an S-adenosyl-L-methionine (SAM)-dependent cytoplasmatic enzyme that is involved in the hepatic biotransformation and detoxification of many drugs and xenobiotics. It is a phase II drug-metabolising enzyme that catalyses the N-methylation of nicotinamide and other structurally related pyridinyl compounds [6]. NNMT was first identified in liver [7] and it has been reported to be overexpressed in several tumors [8,9,10]. Interestingly, NNMT upregulation has been correlated with enhanced cancer cell migration and invasion, promoting the epithelial-mesenchymal transition (EMT) [11,12], and with adverse clinical outcomes [13,14]. Moreover, the NNMT expression affects cellular resistance to radiation-induced damage [15,16,17] and the tumoral sensitivity to anti-tumor agents [18], suggesting that it could play a pivotal role in cancer cell metabolism. Finally, high NNMT levels were also detected in urinary samples of bladder and oral cancer patients [19,20], suggesting that its presence in biological fluids could be used as a marker for the early diagnosis of these malignancies. Given the evidence that NNMT contributes to carcinogenesis, this report aims to conduct a comprehensive narrative review of all of the available literature regarding the biological, diagnostic, and prognostic role of NNMT in HNT.

## 2. Oral Squamous Cell Carcinoma

The oral squamous cell carcinoma (OSCC) is the most common malignancy of the head and neck region, representing up to 90% of oral cavity cancers [21]. Annually, over 400,000 new cases are diagnosed worldwide, and the annual incidence was higher in southern Asia, with age-standardized incidence rates of >10 cases/100,000 population per year [22]. OSCC can arise at any age and may affect any anatomical oral site; however, it mainly affects adult males over 50 years-old and the tongue represents the most involved oral subsite [23]. There are several risk factors, such as alcohol consumption, tobacco and betel nut use, and HPV infection that are strongly related to the development of OSCC; moreover, OSCC can arise from potentially malignant disorders or directly from apparently normal mucosa [24]. It is characterised by a high incidence of locoregional recurrence and lymph node metastasis, with a five-year survival rate that is equal to 66.9%. Among the head and neck tumors, OSCC is by far the most investigated malignancy regarding the expression of NNMT. Indeed, a number of studies were conducted to evaluate the role of NNMT in oral carcinogenesis.

In 2007, Emanuelli et al. investigated for the first time the NNMT expression in 22 surgical samples of OSCC, with (N+) and without (N0) lymph node metastases, and in corresponding normal mucosa [25]. Real-time quantitative PCR and Western blot analysis found increased levels of NNMT in tumor samples compared to normal tissues, with fold change values ranging between 1.1 and 38 (mean value 7.5, median 3.8). Interestingly, the NNMT levels were higher in the N0 OSCC group than in N+ group. Immunohistochemical analysis confirmed these results, showing a marked increase in NNMT expression in the N0 OSCC group compared to N+ tumors and to normal tissues. Moreover, the tumor size and pathological staging showed an inverse association with NNMT expression. Survival analysis suggested a trend for the relationship between NNMT expression and a good prognosis, but it did not reach statistical significance, possibly due to the small sample size.

Increased levels of NNMT in OSCC was confirmed by Roberg et al., who investigated the genetic and phenotypic characteristics of a tongue squamous cell carcinoma cell line with terminal differentiation deficiency [26]. In this study, the authors established a new OSCC cell line (LK0412) that was capable of replicating in serum-free medium originally devised for culture of normal oral keratinocytes. These cells showed moderate morphological differentiation as well as several changes in cancer-associated genes, including NNMT. In particular, a microarray analysis reported that NNMT is one of the most increased genes, with a 74-fold change increase compared to normal oral keratinocytes.

To investigate the biological role of NNMT in influencing oral cancer cell metabolism, a study was conducted to determine the effect of RNA interference-mediated downregulation of NNMT on OSCC cell proliferation [27]. Its expression was evaluated in OSCC cell lines (PE/CA PJ-15, PE/CA PJ-34, PE/CA PJ-41, PE/CA PJ-46, PE/CA PJ-49, HSC-2, and HSC-3), showing highest NNMT expression levels in PE/CA PJ-15. Therefore, this cell line was selected to be transfected with plasmids that coded short hairpin RNAs against NNMT, showing downregulation at both the mRNA and protein levels. The effects of NNMT gene silencing were a reduction in cell proliferation and colony formation ability on soft agar. Moreover, transfected and control PE/CA PJ-15 cells (5 × 10^6^ cells) were injected into athymic BALB/c nude (nu/nu) mice to investigate the effect of NNMT downregulation on tumor growth. NNMT silencing induced a significant reduction in tumor volume (222.3 ± 65.5 mm^3^ vs. 981.7 ± 137.5 mm^3^), showing that the downregulation of this protein in OSCC inhibits tumorigenicity in vivo.

Recently, the same group confirmed these results by investigating the effects of plasmid-mediated overexpression of NNMT in the OSCC cell line, HSC-2 [28]. This cell line was transfected with a NNMT expression vector, showing upregulation at the mRNA and protein levels, as well as catalytic activity. The effects of transfection on cell proliferation were assessed by a MTT colorimetric assay, showing a significantly increased cell growth in vitro. Moreover, an association between NNMT and survivin-ΔEx3 isoform was found, suggesting a possible role of NNMT in regulating apoptosis-related proteins.

Regarding the clinical application of NNMT as a biomarker for oral cancer, a retrospective prognostic study was conducted in a cohort of 92 specimens with OSCC and 51 surgical samples with normal mucosa with the aim to evaluate the prognostic role of NNMT [29]. A semiquantitative score to assess NNMT expression was used, showing absent or very weak cytoplasmic expression of NNMT in most cases of normal mucosa, with occasionally nuclear staining. On the contrary, NNMT immunostaining was significantly increased in tumor tissue compared to the normal mucosa (*p* = 0.0014), showing an inverse relationship between protein expression and histological grading (*p* = 0.0498). Despite these findings, survival analysis failed to demonstrate an evident prognostic significance of NNMT in oral cancer.

In 2012, the same group conducted a pilot study with the aim to develop a noninvasive diagnostic test for early-stage OSCC [19]. In particular, 27 paired tumor and non-tumor tissue samples from patients with OSCC were investigated to measure the levels of NNMT activity. Compared to the non-tumor tissues, NNMT activity was significantly higher in OSCC (3.322 ± 2.215 vs. 0.6600 ± 1.094; *p* < 0.0001). Moreover, survival analysis found an increase of NNMT enzyme activity from non-tumor to tumor tissues (*p* < 0.0001) and from N0 OSCC to N+ OSCC (*p* < 0.0001). Subsequently, a Western blot analysis was used to evaluate NNMT protein levels in saliva samples that were obtained from 16 patients with OSCC and 15 healthy subjects. The results were promising, showing higher salivary NNMT expression levels in OSCC patients than in healthy subjects (*p* < 0.0001).

In the last two decades, high-throughput sequencing methods have been developed, representing a leap toward the characterization of genomic, transcriptomic, and proteomic expression profiling of the OSCC. In a study that was focused on chewing-tobacco-related OSCC, whole genome expression profiling was conducted in a cohort of patients with the aim to identify new biomarkers [30]. A total of 30 OSCC surgical samples and 27 normal oral tissues were selected for whole genome expression using high-throughput technology. The results showed that 255 overexpressed and 170 downregulated genes, being 20.4% and 25% of which, respectively, related to cell metabolism functions. Interestingly, NNMT was overexpressed in tumor tissue, with a mean 2.39 increase of the transcripts in the OSCC tissues as compared to the normal mucosa, indicating an important role for this gene in oral carcinogenesis (*p* < 0.05). However, no significant associations with clinicopathological data were found, mainly due to the small sample size.

Similarly, a bioinformatic analysis based on affymetrix microarray data investigated differentially expressed genes between eight OSCC samples and one sample of mucosa, as a control group [31]. A total of 372 and 305 genes were overexpressed and downregulated, respectively, and NNMT resulted to be overexpressed (more than a one-fold change) compared to normal oral mucosa (*p* < 0.05). These results were interesting, showing that several differentially expressed genes, including NNMT, are tightly associated with OSCC.

Finally, in 2011 a study conducted a proteomic profiling of 10 advanced-stage OSCC samples with their adjacent non-tumor mucosa to identify differentially expressed proteins [32]. A comparative analysis reported that 17 proteins were differentially expressed in OSCC, and an immunohistochemical analysis on a cohort of 38 OSCC specimens was subsequently conducted. Of these, only the immunohistochemical expression of three proteins, including NNMT, confirmed the proteomic data, although their prognostic role was not clear due to small sample size.

## 3. Upper Aerodigestive Tract Cancer

Nasopharyngeal carcinoma (NPC) is the most common nasopharyngeal cancer and it shows remarkable geographic differences in incidence. It is uncommon among Caucasian, whereas it is an endemic malignancy in Southeast Asia and southern China [2]. Its carcinogenesis is a multi-step process involving many etiological factors. It is widely accepted that the Epstein–Barr virus (EBV) infection, environmental factors, and genetic susceptibility are the major risk factors. Despite the advancements in radiotherapy and in concurrent chemotherapy leading to improved locoregional control, the distant metastasis remains the main cause of death [33].

The NNMT expression in NPC was evaluated in 124 patients that were treated by radiation therapy and/or chemotherapy [33]. The NNMT expression was detected in all of the NPCs and lightly observed in normal nasopharyngeal mucosa. Moreover, high NNMT expression was significantly associated with faster incremental growth (*p* = 0.003) and advanced pathological stage (*p* = 0.006). Following analysis of the transcriptomic profiles of 31 NPCs and 10 normal nasopharyngeal tissues, NNMT was found to be significantly upregulated in advanced stage NPCs compared to the low stage group (*p* = 0.0036). Furthermore, a positive correlation between NNMT and pAkt expression was detected (*p* < 0.001), and higher NNMT and pAkt levels were significantly associated with worse disease-specific survival (DSS) (*p* = 0.0004; *p* = 0.0125), metastasis-free survival (MeFS) (*p* = 0.008; *p* = 0.0063), and local recurrence-free survival (LRFS) (*p* = 0.005; *p* = 0.0125). However, 70% of the enrolled samples were represented by advanced stage tumors, leading to a possible selection bias. Finally, following multivariate analysis, high NNMT expression represented an independent prognostic factor of worse DSS (*p* = 0.02, HR = 1.976) and MeFS (*p* = 0.029, HR = 2.022). Therefore, NNMT overexpression could be related to tumor cell migration and cancer invasiveness in high-stage NPCs, and an aberrant activation of the pAkt pathway may contribute to tumoral progression. Moreover, the pAkt signaling pathway seems to have a significant role in tumoral radio-resistance [16]. It is suggested that the inhibition of PI3K/Akt signaling decreases the binding of metalloproteinase-2 (MMP-2) promoter that is mediated by NNMT. Therefore, NNMT could promote the activation of MMP-2 via a pathway that sequentially involves pAkt signaling.

Laryngeal cancer is the second respiratory tract tumor after lung cancer while laryngeal squamous cell carcinoma (LSCC) is the most common malignancy of the larynx. It occurs mainly in adult males with tobacco habits and alcohol abuse. Other factors, such as gastro-oesophageal reflux, diet, and nutritional factors have been linked to an increased risk of LSCC. Moreover, HPV infection was detected in 4–15% of cases. Supraglottic and subglottic LSCC have the highest risk of lymph node metastasis. The overall five-year survival rate depends on the tumor localization, the outcome is poorer for subglottic and tracheal SCC (25–40%), and on the pathological stage, and is poorer for advanced malignancy (30–40%) [2].

Emanuelli et al. explored the role of NNMT in cancer cell metabolism on a human laryngeal cancer cell line (KB cell line) and the effect of NNMT RNA interference-mediated downregulation on cell proliferation [34]. High values of NNMT mRNA and protein levels as well as a high NNMT immunoreactive protein intensity were detected in KB cancer cells. Moreover, NNMT enzyme activity was higher in the KB cells compared to the control cells that were transfected only by reagent (mock cells), (mean specific activity 1.7 U/mg vs. 0.4 U/mg). Compared to the mock cells, both NNMT mRNA and the protein levels decreased after transfection. In particular, the mean NNMT mRNA expression was 3.33-fold lower in the transfected compared to the mock cells. In the transfected cells, the NNMT downregulation resulted in markedly reduced cell proliferation and growth within 48 h after transfection. The cellular colonies that arose from the NNMT silenced cells were much smaller and fewer with respect to those that originated from the mock cells. These data support the involvement of NNMT in tumor proliferation. The growth of the KB cells since the treatment with vectors that coded shRNA targeted against NNMT efficiently suppressed the gene expression and exhibited a clear inhibitory effect on cell growth. Therefore, NNMT could represent a pivotal molecule that is associated with the cell proliferation and is involved in the preservation of cell viability.

Esophageal squamous cell carcinoma (ESCC) is one of the most common aggressive malignant tumors of the digestive system worldwide. It is characterized by a high degree of malignancy, early nodal and distant metastasis, and a poor prognosis. In particular, the overall five-year survival rate is less than 25%, due to the lack of early diagnosis and ineffective treatment for advanced cancers [13].

The immunohistochemical expression of NNMT in ESCC was investigated in 30 ESCC surgical samples and on corresponding adjacent normal tissues. The NNMT expression in ESCC was significantly higher (*p* < 0.05) in all of the ESCC samples compared to the adjacent normal tissues (100% vs. 23.3%, respectively). Moreover, the majority (73.3%) of NNMT-positive ESCC samples exhibited a strong staining, whereas all the NNMT-positive healthy samples showed low staining intensity. Notably, the NNMT expression significantly correlated with lymph node metastasis (*p* = 0.046). Therefore, these data could suggest that NNMT overexpression may be involved in the carcinogenesis and progression of human ESCC. The authors also evaluated the biological behaviour of NNMT [13] and its effect on 5-fluorouracil (5-FU) sensitivity [18] in several human ESCC cell lines (EC1, EC9706, TE1, TE13, and Eca109 cell lines). NNMT expression was found to be significantly higher in EC9706 and TE1 cells compared to the other cells lines (*p* < 0.05). Silencing the NNMT expression in EC9706 and TE1, led to a 90% decrease in NNMT mRNA and protein levels in both of the cell lines (*p* < 0.05). Moreover, its downregulation significantly suppressed the tumor cell growth, promoting the expression of apoptosis-related proteins (Bax and cleaved-Caspase 3) and decreased the cell proliferation and migration compared to the untreated group (no transfection) and the negative control (NC) group (cells transfected with NC-siRNA sequences). Moreover, the downregulation of mesenchymal transition markers, such as Vimentin, N-cadherin, β-catenin, and *p*-GSK3β, the overexpression of E-cadherin, and the inhibition of Wnt/β-catenin signaling in siNNMT cells, seems to suggest the critical role of NNMT in ESCC metastasis.

Regarding the role of NNMT in the chemotherapy of ESCC, its expression showed a negative association with 5-FU cells sensitivity [18]. The NNMT silencing enhanced the 5-FU inhibitory effect on cell viability and colony formation and increased the 5-FU-induced proapoptotic protein levels in the TE1 cells. The half maximal inhibitory concentration of 5-FU in TE1-siNNMT cells was significantly lower compared to the TE1-NC cells, as well as the glucose consumption and lactate production. Tumors that originated from the transplantation of TE1-siNNMT cells in nude mice were significantly smaller and more sensitive to 5-FU than those that were formed by TE1-NC cells. In TE1-siNNMT tumors, 5-FU promoted the apoptosis and tumoral necrosis. Therefore, the inhibitory effect of 5-FU on tumor growth was significantly enhanced when NNMT was downregulated. In TE1-NC tumor, the knockdown of NNMT, and even more with the 5-FU treatment, significantly decreased the expression of glycolysis-related enzymes. These results suggested that NNMT may decrease the 5-FU sensitivity of the ESCC cells by promoting glucose metabolism which is closely correlated with cancer cell drug resistance. Therefore, the NNMT knockdown could improve the clinical outcomes of chemotherapy in ESCC patients.

## 4. Other Tumors of Maxillo-Facial Region

The role of the NNMT enzyme as a potential tumoral biomarker of salivary glands malignant neoplasms has been evaluated by Mori et al. The authors assessed its biological activity in a lymph node metastatic Adenoid cystic carcinoma (AdCC) cell line expressing green fluorescent protein (ACCS-LN-GFP) that was obtained by an orthotropic transplantation of submandibular metastatic AdCC cells in nude mice [34]. AdCC accounts for <1% of head and neck tumors and <10% of salivary gland neoplasms. It is characterized by invasive growth, perineural invasion, early local recurrence (16–85%), and distant metastasis (25–55%). Radical surgical excision, with or without postoperative radiotherapy, represents the elective therapy. Unfortunately, the five-year survival rate is approximately 35% and local recurrences occur despite combined treatment [2].

The microarray analysis demonstrated that NNMT expression was 2.0 times higher in ACCS-LN-GFP than in ACCS-metastatic-GFP cells (ACCS-M-GFP). Furthermore, the NNMT mRNA and NNMT protein levels of ACCS-M-GFP, and even more of ACCS-LN-GFP, were significantly higher compared to those in the ACCS-GFP cell line [35]. This data supports the critical role of NNMT in AdCC invasion and metastasis. In fact, its expression increased from the low tumorigenic ACCS cell line to the highly metastatic ACCS-M-GFP and to the lymph node metastatic ACCS-LN-GFP cell lines.

Odontogenic tumors (OT) constitute a group of heterogeneous diseases ranging from hamartomatous tissue proliferations to benign and malignant tumors with metastatic potential. The etiopathogenesis are still unknown, most of them arise ex novo while some lesions may originate from pre-existing odontogenic cysts [3]. The NNMT expression was evaluated in 105 surgical samples of odontogenic lesions to assess its role as a prognostic marker of locoregional recurrence [36]. The research focused on ameloblastoma (AM), due to the high frequency of oncogenic alterations that were reported in literature, and on odontogenic keratocyst (OKC), due to its controversial nature. The authors enrolled 55 patients that were affected by primary and recurrent AM (*n* = 50 cases) and OKC (*n* = 55 cases). The cytoplasmatic extension and the staining intensity of NNMT was significantly higher in the recurrent than primary lesions, both in AMs (48.0% vs. 34.6%, *p* = 0.0430; *p* = 0.0470) and in OKCs (*p* = 0.0014; *p* = 0.0276). No statistical differences between NNMT expression and clinicopathological data were found, only a lower NNMT expression was detected on epithelial cells of Acanthomatous AM compared to Follicular AM (*p* = 0.0411). Due to the lack of standardized surgical protocols and the high recurrence rate of odontogenic tumors, the results of this preliminary study are promising, suggesting the potential role of NNMT as prognostic marker of more local aggressiveness and infiltrative lesions.

The same research group investigated the role of NNMT in 30 samples of primary malignant melanoma, comparing oral malignant melanomas (OMM) and cutaneous melanomas (CM) [37]. The OMM is an extremely rare and highly aggressive tumor, accounting for approximately 0.2–8.0% of all melanomas. Most of them show infiltrative invasion at the initial presentation with an overall five-year survival rate ranging from 10 % to 25%. Due to the aggressiveness of OMM, the staging system starts from the T3 category and no established risk factors have been identified. The authors showed that immunohistochemical NNMT extension was significantly higher in the CM group (41.7% vs. 14.6%, *p* = 0.0008), while the NNMT staining intensity was highest in the OMM group (*p* = 0.0253). The NNMT immunoreactivity in the inflammatory infiltrate showed no statistical differences between the two groups. Furthermore, no significant correlations were detected between the NNMT expression and the risk of recurrence in the OMM group. However, the NNMT staining intensity was significantly higher in ulcerated OMM compared to the non-ulcerated OMM group (*p* = 0.044). The presence of higher staining intensity without an increase in NNMT expression in ulcerated OMM could be related to the biological effects of NNMT. In fact, necrotic and peri-necrotic regions of the tumor mass, characterised by a compromised vasculature and reduced nutrient availability, could promote the expression of NNMT. Finally, the overexpression of NNMT negatively affected the disease-free survival (DFS) rate. In particular, OMMs with a high NNMT expression (>10%) showed a lower DFS (*p* = 0.0452).

## 5. Thyroid Cancer

Thyroid carcinoma is the most common endocrine gland malignancy. Papillary thyroid cancer (PTC) accounts for 80% of thyroid carcinomas, followed by follicular carcinoma (10%). A history of radiation exposure represents a well-established environmental factor that is related to PTC and it is also the most prevalent thyroid cancer subtype in countries that have iodine-sufficient or iodine-excess diets. PTC is a well-differentiated tumor that is characterized by a good prognosis with an overall 10-year survival rate equal to 80-90 %. However, the advanced pathological stage, the older age at diagnosis, and the locoregional and distant metastasis are correlated to a poor prognosis [2].

NNMT has been suggested as a potentially useful biomarker in the diagnosis of PTC [38]. Its expression was evaluated in papillary thyroid cancer cell lines (BHP2-7, BHP5-16, BHP 7-13, BHP 10-3, BHP 14-9, BHP 15-3, BHP 17-10, and BHP 18-21), anaplastic cell lines (ARO 81-1 and DRO 90-1), medullary cell lines (HRO 85-1 and DRO 81-1), follicular cell lines (WRO 82-1), and in papillary cell lines (TC1). High levels of NNMT mRNA and high NNMT catalytic activity were detected in all of the papillary cancer cell lines, especially in BHP 2-7, BHP 7-13, BHP 10-3, BHP 18-21, and TPC1, compared to other cancer cell types and thyroid primary cells [38,39]. The immunohistochemical staining of the BHP 2-7 cell line had a stronger extension and intense cytoplasmatic reaction, compared to WRO 82-1 cells. These data were confirmed by immunohistochemistry in 30 specimens of thyroid lesions and 2 healthy tissue samples; all of the PTC groups expressed a stronger cytoplasmic NNMT reaction, while a negative expression was detected in normal tissues and in benign thyroid lesions.

The same authors showed that hepatocyte nuclear factor-1β (HNF-1β) is expressed in papillary cancer cell lines with high NNMT expression and it could be involved in the activation of NNMT transcription [38]. In fact, a higher HNF-1 β expression was detected in papillary cell lines with high NNMT activity (BHP 2–7, BHP 7–13, BHP 10–3, BHP 18–21, and TPC1). Its expression level was significantly higher even compared to a liver cancer cell line (Hep G2 cells); whereas other papillary cell lines with negative or lower NNMT gene expression, resulted HNF-1β negative. In the BHP 2–7 cells, the HNF-1β binding site mutation, identified in the NNMT promotion region, decreased NNMT promoter activity about five-fold. This effect was significantly lower and negative in Hep G2 cells and in NNMT negative cell lines, respectively. Therefore, the putative HNF-1β site is functional for NNMT promoter activity and can activate the NNMT promoter efficiently in the absence of the upstream regulatory elements. However, the involvement of other factors binding to the upstream NNMT sequence cannot be excluded. Furthermore, the authors demonstrated that depsipeptide, a bicyclic peptide with anticancer activity, downregulated NNMT and HNF-1β gene expression in the BHP 18-21 cell line. The inhibitory effect of depsipeptide on NNMT and HNF-1β gene expression was found to be time-dependent. In particular, their mRNA and protein levels started to significantly decrease after 16 h of treatment. These results suggested that the NNMT gene downregulation is a selective repression that is occurring at the transcriptional level. It is worth noting that the HNF-1β site in the NNMT basal promoter region was required for repression of NNMT promoter activity by depsipeptide. These results suggest that the repression of HNF-1β is partially responsible for the depsipeptide-mediated repression of NNMT gene expression [40].

## 6. Discussion

**Figure 1:**
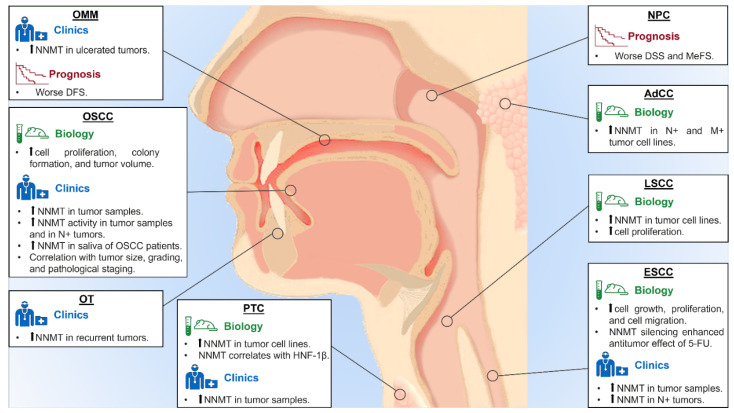
Main findings on the role of NNMT in HNT. OSCC: oral squamous cell carcinoma; AdCC: adenoid cystic carcinoma; OMM: oral malignant melanoma; OT: odontogenic tumor; PTC: papillary thyroid carcinoma; NPC: nasopharyngeal carcinoma; LSCC: laryngeal squamous cell carcinoma; ESCC: esophageal squamous cell carcinoma; IHC: immunohistochemistry; DFS: disease-free survival; DSS: disease-specific survival; MeFS: metastasis-free survival; HNF-1β: hepatocyte nuclear factor-1-beta; 5-FU: 5-fluorouracil.

NNMT is the only enzyme that is able to use nicotinamide as a methyl acceptor substrate and is the main regulator of intracellular nicotinamide levels. Therefore, NNMT expression could affect the cellular events that involve nicotinamide, modulating its excretion after N-methylation [9]. NNMT is strongly expressed in liver, with significant differences between individuals [7]. Its upregulation has been described in many solid malignancies including HNT, although the biological effects of NNMT expression and activity in cancer cell metabolism remains unclear [8,10]. Given the evidence that NNMT contributes to carcinogenesis, several studies have been conducted to explore the role of this protein in NHT (Table 1, Figure 1).

Based on immunohistochemistry, the NNMT protein expression showed to be significantly upregulated within tumor tissues. In particular, NNMT expression is higher in OSCC tissue compared to normal mucosa [25], while its expression seems to decrease with increasing histological grading, tumor size, lymph node metastasis, and pathological staging [25,29]. This trend of NNMT expression in OSCC suggests that this protein may be significant in early stages of malignant progression. On the contrary, NNMT overexpression in NPC and ESCC is associated with higher tumor size, advanced pathological staging, and the presence of lymph node metastasis, suggesting a role in cancer cell migration and invasiveness [13,33]. Similar results are reported in PTC, showing higher cytoplasmatic NNMT expression compared to normal tissue and benign thyroid lesions [39]. The NNMT upregulation in recurrent benign odontogenic lesions emphasizes the intrinsic growth potential of the odontogenic epithelium, suggesting the presence of different features that underly metabolism and cell proliferation activity [36]. Finally, in ulcerated OMM, the higher NNMT staining intensity could be related to its biological effects. Indeed, necrotic and peri-necrotic regions of the tumor mass that were characterized by compromised vasculature and reduced nutrient availability, could promote the expression of NNMT [37]. Some studies reported the occasional presence of nuclear staining of NNMT both in normal and cancer tissues, although NNMT is described to be a cytoplasmic protein [25,39]. Since almost all of the immunohistochemical studies used a polyclonal antibody, the presence of nuclear staining could be related to an antibody cross-reaction with unknown nuclear components present at higher levels. Therefore, the nature of the NNMT nuclear staining needs to be further studied [41]. Immunohistochemical analyses also suggest a different biological impact of NNMT on cancer cell metabolism depending on the HNT subtype, since its expression appears to be higher in the early or advanced stages in different tumor types.

Several studies that investigated the activity and the biological role of NNMT, found high NNMT mRNA and protein levels and high catalytic activity in different cancer cell lines, including LSCC cell lines [34], ESCC cell lines EC9706 and TE1 [13], and PTC cell line BHP 2-7 [39]. Moreover, NNMT upregulation from low tumorigenic to the highly metastatic AdCC cell lines was demonstrated [35]. These studies also explored the involvement of NNMT on modulating cancer cell proliferation. The shRNA-mediated silencing of NNMT efficiently suppressed gene expression and exhibited a clear inhibitory effect on in vitro cell proliferation and growth, promoting the expression of apoptosis-related proteins, and modulating the cell cycle [13,27,34]. These findings suggested that NNMT knockdown could exert antitumor effects, representing a pivotal molecule that is involved in the cell viability preservation and a potential molecular target for cancer therapy. Moreover, the downregulation of mesenchymal transition markers, the overexpression of epithelial markers, and the inhibition of Wnt/β-catenin signalling in sh-NNMT cells of ESCC, suggests the critical role of NNMT in metastasis. In particular, NNMT could modulate EMT and cell migration via Wnt/β-catenin signalling pathway [13]. The association between NNMT and pAkt expression could be related to cancer cell migration and invasiveness in high-stage NPCs. In addition, an aberrant activation of the pAkt pathway seems to have a significant role in tumoral radio-resistance, since the inhibition of PI3K/Akt signalling decreases the binding of MMP-2 promoter that is mediated by NNMT [33]. The HNF-1β upregulation in high-NNMT TCP cell lines suggests that it could be involved in the activation of NNMT transcription. Indeed, the putative HNF-1β site seems to activate the NNMT promoter even in the absence of upstream regulatory elements [40]. In hepatocytes, the NNMT regulates glucose metabolism and enhanced its disposal. Since the glucose metabolism is closely related with cancer drug resistance and considering the association between NNMT upregulation and the 5-FU effects on cell proliferation, NNMT may regulate 5-FU sensitivity via the Warburg effect in ESCC, promoting glucose consumption and lactate production. These data are corroborated by the results that were obtained in vivo from sh-NNMT xenograft on nude mice, showing that knockdown of NNMT could significantly increase the sensitivity of ESCC cells to 5-FU and suppress the Warburg effect in vivo [18].

The relationship between NNMT overexpression and adverse clinical outcomes is supported by several studies linking enhanced cell proliferation, decreased apoptosis, and radiation and chemoradiation resistance to overexpressed NNMT levels [9,11,12,14,15,39,42,43,44]. Indeed, many studies agree in showing that NNMT overexpression is associated with cell viability and proliferation, cell migration, colony formation ability, and tumor growth in animal models [13,27,34]. Moreover, higher NNMT expression is associated with stage and grade progression in several tumor types [25,29]. However, these results contrast with those regarding the prognostic role of NNMT in HNT. Indeed, NNMT seems to be an independent prognostic factor of worse DSS and MeFS in PTC [33], while several studies failed to demonstrate a prognostic significance of this protein in OSCC [19,25]. These results may be due to several methodological limitations in the reported studies, such as the retrospective nature, the inadequate sample size, and the presence of a possible selection bias. In some cases, NNMT might be a potential candidate as diagnostic marker in HNT. Indeed, NNMT expression may be useful in the differential diagnoses between papillary carcinomas and benign thyroid lesions with papillary architecture [39], or between primary or recurrent odontogenic lesion [36].

In conclusion, carcinogenesis in the head and neck region is a multistep process proceeding from single gene mutations that are generated by carcinogens that have caused substantial dysregulation of metabolic processes. However, the molecular mechanisms underlying HNT are still poorly understood and several molecular and cellular key points still need to be properly investigated. Despite significant advances in cancer research over the last few years, no valid molecular biomarkers that are capable of predicting the biological aggressiveness have been validated so far to stratify HNT patients [45]. Therefore, new, sensitive biomarkers and new targets for molecular-based treatments are necessary to improve the diagnosis and prognosis of HNT. NNMT appears to be a promising candidate as its overexpression is associated with a more aggressive neoplastic phenotype and with stage and grade progression (Table 2). However, further studies on larger cohorts of patients are needed to confirm these findings and to explore the biological role of NNMT in HNT. Moreover, this enzyme could be a promising target for cancer therapy, which can be studied in more detail thanks to the development of specific NNMT inhibitors which have shown promising results in preclinical studies [46,47,48,49,50,51].

## Figures and Tables

**Table 1:** Summary of the literature data on the role of NNMT in HNT.

Author, Year [ref]	Tumor Type	Methods	Materials	Main Results
Sartini D et al., 2007 [25]	OSCC	IHC	Rabbit polyclonal anti-human NNMT Ab, 1:500	NNMT expression in N0 OSCC is higher than N+ OSCC and normal tissues. Inverse association between NNMT expression and tumor size and pathological staging.
Roberg K et al., 2008 [26]	OSCC	Genomic study	Genomic microarray [Affymetrix HG-Focus chip] (LK0412 cell line)	NNMT expression is 74-fold change higher in OSCC than in normal oral keratinocytes.
Emanuelli M et al., 2010 [29]	OSCC	IHC	Rabbit polyclonal anti-human NNMT Ab, 1:500	NNMT immunostaining is higher in tumor tissue compared to normal mucosa. Moreover, there is an inverse relationship between NNMT expression and histological grading.
Liao KA et al., 2011 [32]	OSCC	Proteomic study	Liquid chromatography-mass spectrometry [LTQ-Orbitrap hybrid tandem mass spectrometer and Agilent 1200 nanoflow HPLC] (OSCC and adjacent non tumor tissue)	A total of 17 proteins were differentially expressed in OSCC compared to non-tumor tissue, including NNMT.
		IHC	Rabbit polyclonal anti-human NNMT Ab, 1:50	The expression of three proteins, including NNMT, confirmed the proteomic data.
Sartini D et al., 2012 [19]	OSCC	In vitro study	Frozen OSCC tissue samples	Increase of NNMT enzyme activity from non-tumor tissue to OSCC and from N0 to N+ OSCC.
		Clinical study	Saliva from OSCC patients and healthy subjects	Salivary NNMT levels higher in OSCC patients than in healthy subjects.
Pozzi V et al., 2013 [27]	OSCC	In vitro study	PE/CA PJ-15 cell line	NNMT gene silencing reduce cell proliferation and colony formation ability.
		In vivo study	BALB/c nude mice	NNMT silencing reduce tumor volume.
Jiang Q et al., 2014 [31]	OSCC	Bioinformatic study	Bioinformatic analysis based on genomic microarray [Affymetrix Human Genome U133A Array]	NNMT found to be overexpressed (>1-fold change) compared to normal oral mucosa.
Chakrabarti S et al., 2015 [30]	OSCC	Genomic study	Genomic microarray [IlluminaSentrix Human Ref-8 v2 Expression BeadChip arrays] (OSCC patients and healthy subjects)	NNMT expression in OSCC is higher (2.39-fold change) than in normal mucosa.
Seta R et al., 2019 [28]	OSCC	In vitro study	HSC-2 cell line	NNMT overexpression increase cell growth in vitro. Moreover, NNMT is associated to survivin-ΔEx3 isoform expression.
Ishibashi K et al., 2018 [35]	AdCC	In vitro study	ACCS-GFP, ACCS-M-GFP, ACCS-LN-GFP cell lines	NNMT mRNA and protein levels increase from ACCS-GFP line to ACCS-M-GFP and to ACCS-LN-GFP cell line.
		Genomic study	Genomic microarray [60K Agilent 60-mer oligomicroarray] (ACCS-M-GFP, ACCS-LN-GFP cell lines)	NNMT expression is higher (2.0-fold change) in ACCS-LN-GFP than in ACCS-M-GFP.
Mascitti M et al., 2019 [37]	OMM	IHC	Rabbit polyclonal anti-human NNMT Ab, 1:1500	NNMT intensity is higher in ulcerated OMM compared to no-ulcerated OMM. NNMT overexpression negatively affects the DFS.
Mascitti M et al., 2020 [36]	OT	IHC	Rabbit polyclonal anti-human NNMT Ab, 1:1500	NNMT expression is higher in recurrent than primary lesions.
Xu J et al., 2003 [39]	thyroid cancer	In vitro study	BHP2-7, BHP5-16, BHP 7-13, BHP 10-3, BHP 14-9, BHP 15-3, BHP 17-10, BHP 18-21, ARO 81-1, DRO 90-1, HRO 85-1, DRO 81-1, WRO 82-1, TC1 cell lines	NNMT mRNA and catalytic activity are higher in BHP 2-7, BHP 7-13, BHP 10-3, BHP 18-21 and TPC1, compared to other cancer cell types and thyroid primary cells (higher NNMT expression detected on BHP 2-7 cell line).
		IHC	Rabbit polyclonal anti-human NNMT Ab, 1:3000	PTCs overexpress NNMT, normal tissues and benign thyroid lesions do not express NNMT.
Xu J et al., 2005 [38]	PTC	In vitro study	BHP 2-7, BHP 7-13, BHP 10-3, BHP 18-21, TPC 1, NPA 87, BHP 5-16, BHP 14-9, BHP 15-3, WRO 82-1, ML-1A, ML-1B, FTC133, FTC238, XTC-1, O4 PC, HX5 PC, Hep G2, LNCaP, MCF-7 cell lines	Higher HNF-1β expression is detected in papillary cell lines with high NNMT activity.
Xu J et al., 2006 [40]	PTC	In vitro study	BHP 18-21, BHP 2-7, BHP 14-9, Hep G2 cell lines	The HNF-1β binding site mutation significantly decreases the NNMT promoter activity in BHP 2-7 cells compare to Hep G2 cells. The depsipeptide downregulates NNMT and HNF-1β gene expression in BHP 18-21 cell line.
Win T et al., 2013 [33]	NPC	IHC	Mouse monoclonal anti-human NNMT Ab, 1:200	NNMT overexpression is an independent prognostic factor of worse DSS and MeFS.
Pozzi V et al., 2011 [34]	LSCC	In vitro study	KB cell line	KB cells show higher NNMT expression levels compare to mock cell. NNMT levels significantly decreases after silencing. NNMT downregulation significantly reduces the cell proliferation.
Cui Y et al., 2019 [13]	ESCC	IHC	Rabbit polyclonal anti-human NNMT Ab, 1:75	NNMT expression is significantly higher in ESCCs compared to the adjacent normal tissues. NNMT overexpression significantly correlated with lymph node metastasis.
		In vitro study	EC1, EC9706, TE1, TE13, Eca109 cell lines	EC9706 and TE1 cell lines show highest NNMT expression. NNMT silencing suppresses tumor cells growth, proliferation, and migration.
Cui Y et al., 2020 [18]	ESCC	In vitro study	EC1, TE1, Eca109 cell lines	NNMT silencing enhances 5-FU inhibitory effect on cell viability and colony formation and increases 5-FU-induced proapoptotic protein levels in the TE1 cells. TE1-siNNMT tumors are significantly smaller and more sensitivity to 5-FU respect to TE1-NC tumors. In TE1-siNNMT tumors, the 5-FU promotes the apoptosis and the tumoral necrosis.
		Metabolomic study	Gas chromatography-mass spectrometry [Agilent Technologies] (EC1, TE1, Eca109 cell lines)	Nicotinate and nicotinamide metabolism and tricarboxylic acid cycle in TE1 cells are significantly different from those in EC1 and Eca109 cells.

OSCC: oral squamous cell carcinoma; AdCC: adenoid cystic carcinoma; OMM: oral malignant melanoma; OT: odontogenic tumor; PTC: papillary thyroid carcinoma; NPC: nasopharyngeal carcinoma; LSCC: laryngeal squamous cell carcinoma; ESCC: esophageal squamous cell carcinoma; IHC: immunohistochemistry; DFS: disease-free survival; DSS: disease-specific survival; MeFS: metastasis-free survival; HNF-1β: hepatocyte nuclear factor-1-beta; 5-FU: 5-fluorouracil; NC: negative control; siNNMT: silenced NNMT.

**Table 2:** Main findings regarding the role of NNMT in HNT.

Tumor Type	Biological and Clinical Role of NNMT
OSCC	- Increase in cell proliferation, colony formation, and tumor volume; - Overexpression in tumor samples; - Association with tumor size, histological grading, and pathological staging; - Higher enzyme activity in N+ OSCC; - Higher salivary levels in OSCC patients.
AdCC	- Higher expression in N+ and M+ AdCC cell lines.
OT	- Overexpression in recurrent lesions.
OMM	- Overexpression in ulcerated OMM; - Association with worse DFS.
PTC	- Overexpression in PTC samples and cell lines; - Correlation between NNMT and HNF-1β overexpression.
NPC	- Association with worse DSS and MeFS.
LSCC	- Overexpression in cell lines; - Increase in cell proliferation.
ESCC	- Overexpression in tumor samples - Overexpression in N+ ESCC; - Increase in cells growth, proliferation, and migration; - Increase tumor cell viability.

## References

[1] Bernier J. (2016). Head and Neck Cancer: Multimodality Management.

[2] El-Naggar A.K., Chan J.K.C., Grandis J.R., Takata T., Slootweg P.J. (2017). WHO Classification of Head and Neck Tumours.

[3] Mascitti M., Togni L., Troiano G., Caponio V.C.A., Sabatucci A., Balercia A., Rubini C., Lo Muzio L., Santarelli A. (2020). Odontogenic tumours: A 25-year epidemiological study in the Marche region of Italy. Eur. Arch. Otorhinolaryngol..

[4] Amin M.B., Edge S.B., Greene F.L., Byrd D.R., Brookland R.K., Washington M., Gershenwald J., Compton C., Hess K., Sullivan D.C. (2017). AJCC Cancer Staging Manual.

[5] Sandulache V.C., Myers J.N. (2012). Altered metabolism in head and neck squamous cell carcinoma: An opportunity for identification of novel biomarkers and drug targets. Head Neck.

[6] Van Haren M.J., Sastre Torano J., Sartini D., Emanuelli M., Parsons R.B., Martin N.I. (2016). A Rapid and Efficient Assay for the Characterization of Substrates and Inhibitors of Nicotinamide N-Methyltransferase. Biochemistry.

[7] Rini J., Szumlanski C., Guerciolini R., Weinshilboum R.M. (1990). Human liver nicotinamide N-methyltransferase: Ion-pairing radiochemical assay, biochemical properties and individual variation. Clin. Chim. Acta.

[8] Jung J., Kim L.J., Wang X., Wu Q., Sanvoranart T., Hubert C.G., Prager B.C., Wallace L.C., Jin X., Mack S.C. (2017). Nicotinamide metabolism regulates glioblastoma stem cell maintenance. JCI Insight.

[9] Sartini D., Muzzonigro G., Milanese G., Pierella F., Rossi V., Emanuelli M. (2006). Identification of nicotinamide N-methyltransferase as a novel tumor marker for renal clear cell carcinoma. J. Urol..

[10] Xie X., Yu H., Wang Y., Zhou Y., Li G., Ruan Z., Li F., Wang X., Liu H., Zhang J. (2014). Nicotinamide N-methyltransferase enhances the capacity of tumorigenesis associated with the promotion of cell cycle progression in human colorectal cancer cells. Arch. Biochem. Biophys..

[11] Wu Y., Siadaty M.S., Berens M.E., Hampton G.M., Theodorescu D. (2008). Overlapping gene expression profiles of cell migration and tumor invasion in human bladder cancer identify metallothionein 1E and nicotinamide N-methyltransferase as novel regulators of cell migration. Oncogene.

[12] Tang S.W., Yang T.C., Lin W.C., Chang W.H., Wang C.C., Lai M.K., Lin J.Y. (2011). Nicotinamide N-methyltransferase induces cellular invasion through activating matrix metalloproteinase-2 expression in clear cell renal cell carcinoma cells. Carcinogenesis.

[13] Cui Y., Zhang L., Wang W., Ma S., Liu H., Zang X., Zhang Y., Guan F. (2019). Downregulation of nicotinamide N-methyltransferase inhibits migration and epithelial-mesenchymal transition of esophageal squamous cell carcinoma via Wnt/beta-catenin pathway. Mol. Cell. Biochem..

[14] Ogawa A., Griffin R.J., Song C.W. (2000). Effect of a combination of mild-temperature hyperthermia and nicotinamide on the radiation response of experimental tumors. Radiat. Res..

[15] Kassem H., Sangar V., Cowan R., Clarke N., Margison G.P. (2002). A potential role of heat shock proteins and nicotinamide N-methyl transferase in predicting response to radiation in bladder cancer. Int. J. Cancer.

[16] Li H.F., Kim J.S., Waldman T. (2009). Radiation-induced Akt activation modulates radioresistance in human glioblastoma cells. Radiat. Oncol..

[17] Xia S., Zhao Y., Yu S., Zhang M. (2010). Activated PI3K/Akt/COX-2 pathway induces resistance to radiation in human cervical cancer HeLa cells. Cancer Biother. Radiopharm..

[18] Cui Y., Yang D., Wang W., Zhang L., Liu H., Ma S., Guo W., Yao M., Zhang K., Li W. (2020). Nicotinamide N-methyltransferase decreases 5-fluorouracil sensitivity in human esophageal squamous cell carcinoma through metabolic reprogramming and promoting the Warburg effect. Mol. Carcinog..

[19] Sartini D., Pozzi V., Renzi E., Morganti S., Rocchetti R., Rubini C., Santarelli A., Lo Muzio L., Emanuelli M. (2012). Analysis of tissue and salivary nicotinamide N-methyltransferase in oral squamous cell carcinoma: Basis for the development of a noninvasive diagnostic test for early-stage disease. Biol. Chem..

[20] Sartini D., Muzzonigro G., Milanese G., Pozzi V., Vici A., Morganti S., Rossi V., Mazzucchelli R., Montironi R., Emanuelli M. (2013). Upregulation of tissue and urinary nicotinamide N-methyltransferase in bladder cancer: Potential for the development of a urine-based diagnostic test. Cell Biochem. Biophys..

[21] Siegel R.L., Miller K.D., Jemal A. (2017). Cancer Statistics, 2017. CA Cancer J. Clin..

[22] Fitzmaurice C., Allen C., Barber R.M., Barregard L., Bhutta Z.A., Brenner H., Dicker D.J., Chimed-Orchir O., Dandona R., Global Burden of Disease Cancer Collaboration (2017). Global, Regional, and National Cancer Incidence, Mortality, Years of Life Lost, Years Lived with Disability, and Disability-Adjusted Life-years for 32 Cancer Groups, 1990 to 2015: A Systematic Analysis for the Global Burden of Disease Study. JAMA Oncol..

[23] Mascitti M., Zhurakivska K., Togni L., Caponio V.C.A., Almangush A., Balercia P., Balercia A., Rubini C., Lo Muzio L., Santarelli A. (2020). Addition of the tumour-stroma ratio to the 8th edition American Joint Committee on Cancer staging system improves survival prediction for patients with oral tongue squamous cell carcinoma. Histopathology.

[24] Mascitti M., Tempesta A., Togni L., Capodiferro S., Troiano G., Rubini C., Maiorano E., Santarelli A., Favia G., Limongelli L. (2020). Histological features and survival in young patients with HPV-negative oral squamous cell carcinoma. Oral Dis..

[25] Sartini D., Santarelli A., Rossi V., Goteri G., Rubini C., Ciavarella D., Lo Muzio L., Emanuelli M. (2007). Nicotinamide N-methyltransferase upregulation inversely correlates with lymph node metastasis in oral squamous cell carcinoma. Mol. Med..

[26] Roberg K., Ceder R., Farnebo L., Norberg-Spaak L., Grafstrom R.C. (2008). Multiple genotypic aberrances associate to terminal differentiation-deficiency of an oral squamous cell carcinoma in serum-free culture. Differentiation.

[27] Pozzi V., Sartini D., Morganti S., Giuliante R., Di Ruscio G., Santarelli A., Rocchetti R., Rubini C., Tomasetti M., Giannatempo G. (2013). RNA-mediated gene silencing of nicotinamide N-methyltransferase is associated with decreased tumorigenicity in human oral carcinoma cells. PLoS ONE.

[28] Seta R., Mascitti M., Campagna R., Sartini D., Fumarola S., Santarelli A., Giuliani M., Cecati M., Muzio L.L., Emanuelli M. (2019). Overexpression of nicotinamide N-methyltransferase in HSC-2 OSCC cell line: Effect on apoptosis and cell proliferation. Clin. Oral. Investig..

[29] Emanuelli M., Santarelli A., Sartini D., Ciavarella D., Rossi V., Pozzi V., Rubini C., Lo Muzio L. (2010). Nicotinamide N-Methyltransferase upregulation correlates with tumour differentiation in oral squamous cell carcinoma. Histol. Histopathol..

[30] Chakrabarti S., Multani S., Dabholkar J., Saranath D. (2015). Whole genome expression profiling in chewing-tobacco-associated oral cancers: A pilot study. Med. Oncol..

[31] Jiang Q., Yu Y.C., Ding X.J., Luo Y., Ruan H. (2014). Bioinformatics analysis reveals significant genes and pathways to target for oral squamous cell carcinoma. Asian Pac. J. Cancer Prev..

[32] Liao K.A., Tsay Y.G., Huang L.C., Huang H.Y., Li C.F., Wu T.F. (2011). Search for the tumor-associated proteins of oral squamous cell carcinoma collected in Taiwan using proteomics strategy. J. Proteome Res..

[33] Win K.T., Lee S.W., Huang H.Y., Lin L.C., Lin C.Y., Hsing C.H., Chen L.T., Li C.F. (2013). Nicotinamide N-methyltransferase overexpression is associated with Akt phosphorylation and indicates worse prognosis in patients with nasopharyngeal carcinoma. Tumour Biol..

[34] Pozzi V., Mazzotta M., Lo Muzio L., Sartini D., Santarelli A., Renzi E., Rocchetti R., Tomasetti M., Ciavarella D., Emanuelli M. (2011). Inhibiting proliferation in KB cancer cells by RNA interference-mediated knockdown of nicotinamide N-methyltransferase expression. Int. J. Immunopathol. Pharmacol..

[35] Ishibashi K., Ishii K., Sugiyama G., Sumida T., Sugiura T., Kamata Y.U., Seki K., Fujinaga T., Kumamaru W., Kobayashi Y. (2018). Deregulation of Nicotinamide N-Methyltransferase and Gap Junction Protein Alpha-1 Causes Metastasis in Adenoid Cystic Carcinoma. Anticancer Res..

[36] Mascitti M., Sartini D., Togni L., Pozzi V., Rubini C., Santarelli A., Emanuelli M. (2020). Differential expression of nicotinamide N-methyltransferase in primary and recurrent ameloblastomas and odontogenic keratocysts. Eur. J. Clin. Investig..

[37] Mascitti M., Santarelli A., Sartini D., Rubini C., Colella G., Salvolini E., Ganzetti G., Offidani A., Emanuelli M. (2019). Analysis of nicotinamide N-methyltransferase in oral malignant melanoma and potential prognostic significance. Melanoma Res..

[38] Xu J., Capezzone M., Xu X., Hershman J.M. (2005). Activation of nicotinamide N-methyltransferase gene promoter by hepatocyte nuclear factor-1beta in human papillary thyroid cancer cells. Mol. Endocrinol..

[39] Xu J., Moatamed F., Caldwell J.S., Walker J.R., Kraiem Z., Taki K., Brent G.A., Hershman J.M. (2003). Enhanced expression of nicotinamide N-methyltransferase in human papillary thyroid carcinoma cells. J. Clin. Endocrinol. Metab..

[40] Xu J., Hershman J.M. (2006). Histone deacetylase inhibitor depsipeptide represses nicotinamide N-methyltransferase and hepatocyte nuclear factor-1beta gene expression in human papillary thyroid cancer cells. Thyroid.

[41] Zhang J., Xie X.Y., Yang S.W., Wang J., He C. (2010). Nicotinamide N-methyltransferase protein expression in renal cell cancer. J. Zhejiang Univ. Sci. B.

[42] Sun L.Q., Coucke P.A., Mirimanoff R.O., Buchegger F. (2001). Fractionated irradiation combined with carbogen breathing and nicotinamide of two human glioblastomas grafted in nude mice. Radiat. Res..

[43] Overgaard J., Horsman M.R. (1996). Modification of Hypoxia-Induced Radioresistance in Tumors by the Use of Oxygen and Sensitizers. Semin. Radiat. Oncol..

[44] Droller M.J. (2000). Hypoxic radiosensitizers in radical radiotherapy for patients with bladder carcinoma: Hyperbaric oxygen, misonidazole, and accelerated radiotherapy, carbogen and nicotinamide. J. Urol..

[45] Troiano G., Rubini C., Togni L., Caponio V.C.A., Zhurakivska K., Santarelli A., Cirillo N., Lo Muzio L., Mascitti M. (2020). The immune phenotype of tongue squamous cell carcinoma predicts early relapse and poor prognosis. Cancer Med..

[46] Gao Y., van Haren M.J., Moret E.E., Rood J.J.M., Sartini D., Salvucci A., Emanuelli M., Craveur P., Babault N., Jin J. (2019). Bisubstrate Inhibitors of Nicotinamide N-Methyltransferase (NNMT) with Enhanced Activity. J. Med. Chem..

[47] Neelakantan H., Wang H.Y., Vance V., Hommel J.D., McHardy S.F., Watowich S.J. (2017). Structure-Activity Relationship for Small Molecule Inhibitors of Nicotinamide N-Methyltransferase. J. Med. Chem..

[48] Policarpo R.L., Decultot L., May E., Kuzmic P., Carlson S., Huang D., Chu V., Wright B.A., Dhakshinamoorthy S., Kannt A. (2019). High-Affinity Alkynyl Bisubstrate Inhibitors of Nicotinamide N-Methyltransferase (NNMT). J. Med. Chem..

[49] Chen D., Li L., Diaz K., Iyamu I.D., Yadav R., Noinaj N., Huang R. (2019). Novel Propargyl-Linked Bisubstrate Analogues as Tight-Binding Inhibitors for Nicotinamide N-Methyltransferase. J. Med. Chem..

[50] Gao Y., van Haren M.J., Buijs N., Innocenti P., Zhang Y., Sartini D., Campagna R., Emanuelli M., Parsons R.B., Jespers W. (2021). Potent Inhibition of Nicotinamide N-Methyltransferase by Alkene-Linked Bisubstrate Mimics Bearing Electron Deficient Aromatics. J. Med. Chem..

[51] Gao Y., Martin N.I., van Haren M.J. (2021). Nicotinamide N-methyl transferase (NNMT): An emerging therapeutic target. Drug Discov. Today.

